# Extortion subdues human players but is finally punished in the prisoner’s dilemma

**DOI:** 10.1038/ncomms4976

**Published:** 2014-05-29

**Authors:** Christian Hilbe, Torsten Röhl, Manfred Milinski

**Affiliations:** 1 Evolutionary Theory Group, Max-Planck-Institute for Evolutionary Biology, August-Thienemann-Strasse 2, 24306 Plön, Germany; 2 Program for Evolutionary Dynamics, Harvard University, One Brattle Square, Cambridge, Massachusetts 02138, USA; 3 Department of Evolutionary Ecology, Max-Planck-Institute for Evolutionary Biology, August-Thienemann-Strasse 2, 24306 Plön, Germany

## Abstract

Extortion is the practice of obtaining advantages through explicit forces and threats. Recently, it was demonstrated that even the repeated prisoner’s dilemma, one of the key models to explain mutual cooperation, allows for implicit forms of extortion. According to the theory, extortioners demand and receive an excessive share of any surplus, which allows them to outperform any adapting co-player. To explore the performance of such strategies against humans, we have designed an economic experiment in which participants were matched either with an extortioner or with a generous co-player. Although extortioners succeeded against each of their human opponents, extortion resulted in lower payoffs than generosity. Human subjects showed a strong concern for fairness: they punished extortion by refusing to fully cooperate, thereby reducing their own, and even more so, the extortioner’s gains. Thus, the prospects of extorting others in social relationships seem limited; in the long run, generosity is more profitable.

## Abstract (web summary)

Theory predicts that extortioners, individuals that obtain advantages through forces and threats, can outperform any generous co-player. Here, Hilbe *et al.* show experimentally that humans punish extortion by refusing to cooperate, which reduces the extortioner’s gains, and suggest that generosity is more profitable in the long run.

## Graphical abstract

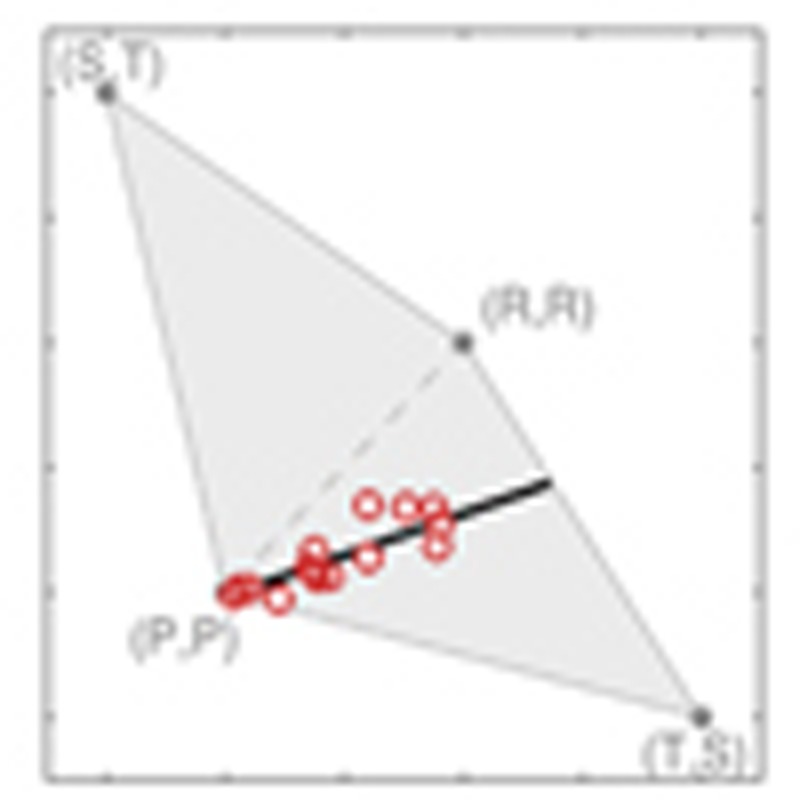

The repeated prisoner’s dilemma has a long tradition of serving as a key model to explore the evolution of cooperation^1^,^2^,^3^,^4^,^5^,^6^. The rules of this stylized game are simple: in each round, two subjects simultaneously decide whether to cooperate or to defect. When both subjects cooperate they each receive a payoff *R*, which exceeds the payoff *P* for mutual defection. However, when a cooperating subject encounters a defector, the defector gets the highest possible payoff *T*, whereas the cooperator obtains the lowest payoff *S*. Although mutual defection is inefficient, it is the unique equilibrium if the prisoner’s dilemma is only played for a single round. However, if subjects have the option to reciprocate past actions in future encounters, a considerable body of evidence suggests that mutual cooperation becomes feasible^7^,^8^,^9^,^10^,^11^,^12^, and that it is in fact favoured by evolutionary forces^13^,^14^,^15^,^16^,^17^,^18^.

Recently, the conclusion that repetition naturally promotes mutual cooperation has been challenged. With an elegant mathematical proof, Press and Dyson^19^ have demonstrated that the repeated prisoner’s dilemma also contains sophisticated strategies that aim to dominate the co-player. Such extortionate strategies have three remarkable properties: (i) they enforce a linear relationship between the player’s own payoff and the opponent’s payoff (strategies with this property were called zero-determinant strategies or ZD strategies); (ii) they prescribe to cooperate sufficiently often, such that the opponent’s best response is to be fully cooperative; (iii) at the same time, extortioners aim to cooperate less often than their opponent, to gain higher payoffs. As a result, extortioners are unbeatable: in a pairwise encounter, they cannot be outperformed by any opponent. These surprising findings have attracted considerable attention^20^, as they suggest that sophisticated players aware of such strategies are able to manipulate and exploit their partners, which should result in an evolutionary advantage.

Despite this relative strength, extortioners have problems to succeed in evolving populations^21^,^22^,^23^. Extortion is unstable: as a homogeneous population of extortioners ends up with the mutual defection payoff *P*, more cooperative strategies can easily invade and take over the population. Eventually, this dynamics may even promote the emergence of generous ZD strategies, which may be considered as the more benevolent counterpart to extortioners^24^. Generous ZD strategies share the first two properties of extortioners: they enforce a linear relationship between the payoffs of the two players, and they provide incentives for the opponent to cooperate. However, as opppsed to extortioners who aim to outcompete their opponents, the payoff of generous players never exceeds the payoff of the co-player. Although generous strategies seem to be too modest to succeed, they evolve under a wide range of conditions^25^,^26^,^27^. Extortionate strategies, on the other hand, require specific assumptions to be successful: extortioners either need to be stubborn and to stick to their strategy^19^, or they need to adopt new strategies at a slower rate than their co-players^21^,^28^,^29^,^30^.

Although these previous theoretical studies offer a fascinating new perspective on direct reciprocity and repeated games, they raise great expectations for studying how the two strategy classes, extortion and generosity, perform against real subjects. To this end, we have designed an economic experiment with four different treatments (see Table 1 and Methods). In each treatment, human subjects played 60 rounds of the prisoner’s dilemma against a predefined computer programme (subjects did not receive any information about the length of the game or the nature of their opponent). The four treatments differed in the implemented ZD strategy of the computer programme, which was either strongly extortionate (ES), mildly extortionate (EM), mildly generous (GM) or strongly generous (GS).

For all treatments, theory predicts that humans maximize their expected payoff by cooperating in every round. In that case, extortioners do not only outperform their human opponents, but they are also expected to receive higher average payoffs than the generous ZD strategies. In the experiment, however, we find that although extortionate strategies indeed dominate their human co-players, this success comes at a cost. Humans are significantly less cooperative against extortioners. As a result, generosity is the more profitable strategy.

## Results

### Performance of ZD strategies against humans

**Figure 1:**
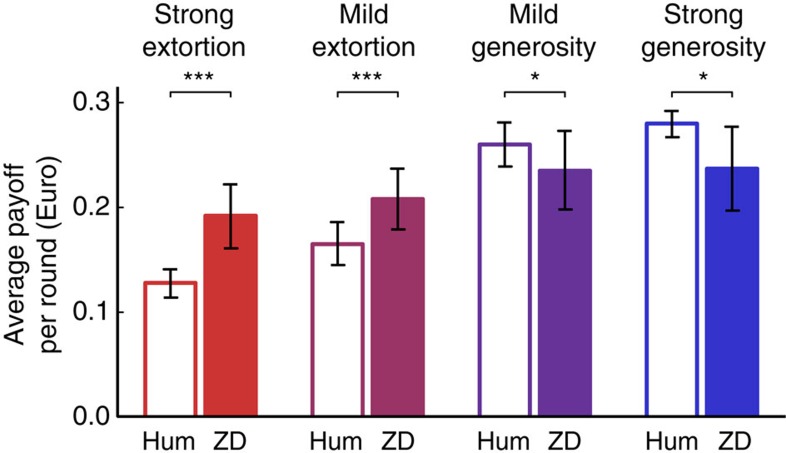
Average payoffs across the four treatments for humans (empty bars) and the ZD strategies implemented by the computer programme (filled bars). In line with the theory, extortioners succeed against their human co-players, whereas generous ZD strategies lag behind their human opponents. Throughout the paper, we use two-tailed non-parametric tests for our statistical analysis, with each iterated game between a human co-player and the computer as our statistical unit (thus we have 16 independent observations for each of the 2 strong treatments, and 14 independent observations for each of the 2 weak treatments). In the above graph, three stars indicate significance at the level *α*=0.001, and one star means significance for *α*=0.05 (using Wilcoxon matched-pairs signed-rank tests with *n*_ES_=*n*_GS_=16, *n*_EM_=*n*_*GM*_=14). As an auxiliary information, we also provide error bars indicating the 95% confidence interval. Individual results for all 60 individuals are presented in the Supplementary Table 1.

Figure 1 shows the resulting average payoffs over all 60 rounds of the game, across the 4 treatments. These results confirm that the two extortionate ZD strategies indeed gain higher payoffs than their human co-players. For example, in the strong extortion treatment, the computer programme obtained an average payoff of *π*_ES_=[euro]0.192 per round, whereas the human subjects earned on average 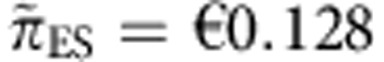 (Wilcoxon matched-pairs signed-rank test, *n*_ES_=16 human co-players, *Z*=3.523, *P*<0.001). Similarly, the mildly extortionate ZD strategy received a payoff of π_EM_=[euro]0.208, which clearly exceeds the mean payoff of the human opponents, 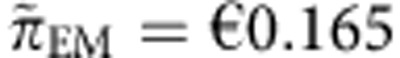 (Wilcoxon matched-pairs signed-rank test, *n*_EM_=14, *Z*=3.181, *P*=0.001).

**Figure 2:**
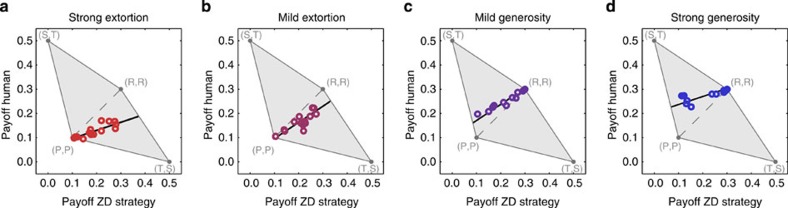
Comparison of experimental results to the theoretical prediction. The grey-shaded area depicts the space of possible payoffs for the two players, that is, the ZD strategy implemented by the computer programme (*x* axis) and the human co-player (*y* axis). The black line corresponds to the theoretical prediction for the expected payoffs (as explained in the Methods) and the open circles indicate the outcome of the experiment. For the extortion treatments (**a**,**b**), these circles are below the diagonal (that is, extortioners outcompete their human co-players), whereas for the generosity treatments (**c**,**d**) these circles are above the diagonal (that is, generous players let their co-players succeed).

Conversely, in the two generosity treatments human subjects had the upper hand, as expected. In the mild generosity treatment, the ZD strategy earned *π*_GM_= [euro]0.235, as compared with the human subjects’ mean payoff 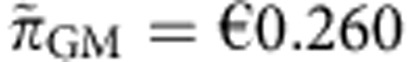 (Wilcoxon matched-pairs signed-rank test, *n*_GM_=14, *Z*=−2.527, *P*=0.012). Lastly, the strong generosity treatment resulted in an average payoff of *π*_GS_=[euro]0.237 for the ZD strategy and 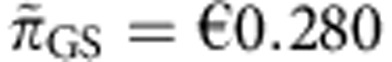 for the human co-players (Wilcoxon matched-pairs signed-rank test, *n*_GS_=16, *Z*=−2.521, *P*=0.012). Thus, extortionate strategies dominated their respective co-players, whereas generous strategies let their co-players succeed. These results are in line with the theory of ZD strategies, which in fact makes virtually no assumptions about human play^19^. In addition, the relationship between the payoffs of the ZD strategist and the human co-player fits reasonably to the linear prediction, as illustrated by Fig. 2, despite the fact that the experimental game is only played for a finite number of rounds (see Methods).

### Comparison of the performance of different ZD strategies

**Figure 3:**
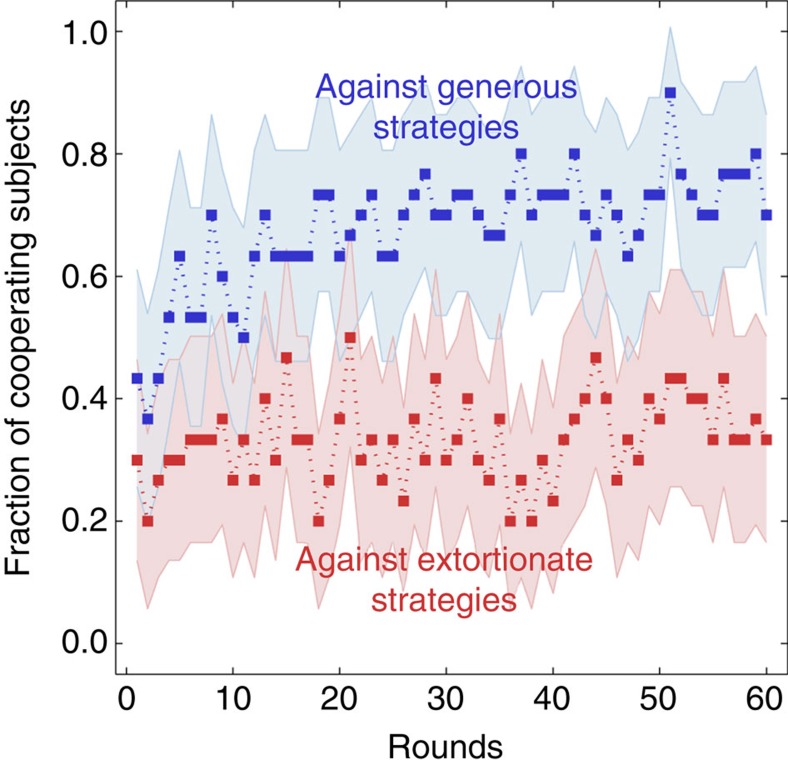
Human cooperation rates over the course of the game. The graph shows the fraction of cooperating human subjects for each round for the two generosity treatments and the two extortion treatments. Dots represent the outcome of the experiment, with the shaded areas depicting the 95% confidence interval. Both curves start with cooperation rates around 30–40%. However, for the generous strategies we find a significant trend towards more cooperation, whereas for the extortionate strategies the average cooperation rates remain stable.

Surprisingly, however, both extortionate ZD strategies yielded a lower payoff than their two generous counterparts. Indeed, when we pool the two extortionate treatments and the two generous treatments, we find that generosity resulted in a >18% increase in payoffs (Mann–Whitney *U*-test, *n*_E_=*n*_G_=30, *Z*=−2.544, *P*=0.011). Against an extortionate ZD strategy, the mean cooperation rate of the human co-players was 34.2%, which is only half of the cooperation rate against generous ZD strategies, 67.7% (Mann–Whitney *U*-test, *n*_E_=*n*_G_=30, *Z*=−3.625, *P*<0.001). This gap comes unexpected, as the different ZD strategies provide similar incentives for their human co-players to cooperate (as indicated by the matching slope values in Table 1). However, a comparison of the human decisions over the course of the game suggests that the treatments followed a different dynamical pattern (Fig. 3). Generous ZD strategies were more successful in motivating their human co-players towards more cooperation: in the two generous treatments, humans had a cooperation rate of 53.0% during the first ten rounds, as compared with 76.0% during the last ten rounds (Wilcoxon matched-pairs signed-rank test, *n*_G_=30, *Z*=3.161, *P*=0.002). In contrast, when paired with an extortionate ZD strategy, the cooperation rate of human subjects only slightly increased from 30.3% during the first ten rounds to 39.7% during the last ten rounds (this increase was not significant, Wilcoxon matched-pairs signed-rank test, *n*_*E*_=30, *Z*=1.131, *P*=0.258).

**Figure 4:**
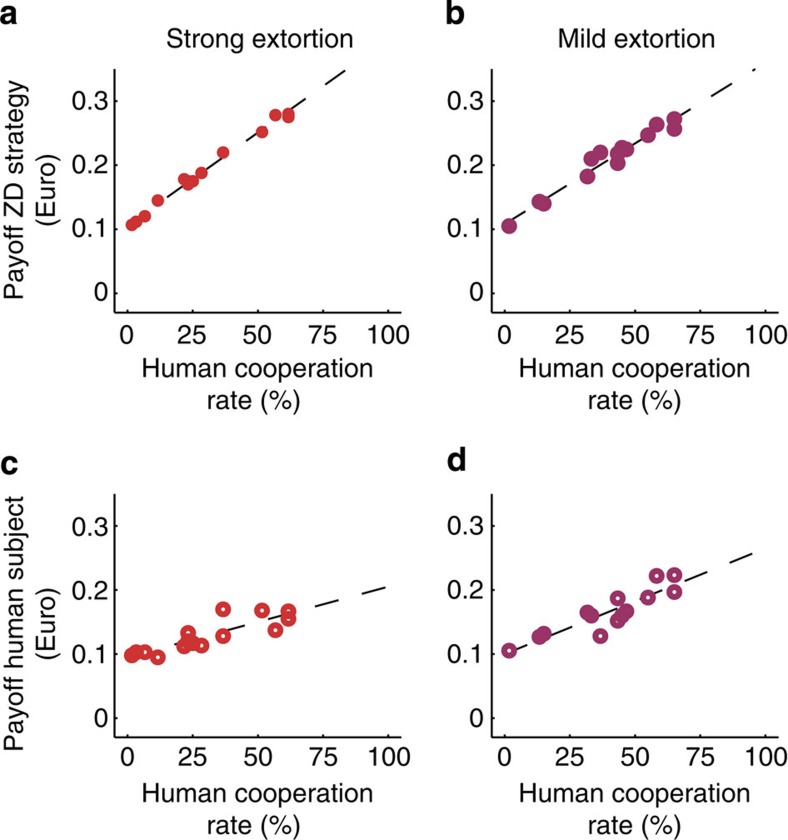
Withholding cooperation as a form of costly punishment. The graph shows the effects of of human cooperation on the payoffs of ZD strategies (**a**,**b**) and on the human subjects’ payoffs (**c**,**d**). The horizontal axis shows the fraction of rounds in which the human players cooperated. Coloured dots represent the outcome of the experiment, whereas the dashed line depicts the linear regression curve based on a least squares analysis. Human cooperation had a strongly positive impact on the co-player’s payoff, and a weakly positive impact on the own payoff. Thus withholding cooperation punishes extortion.

These results suggest that humans were somewhat reluctant to cooperate against extortioners. In fact, in the extortion treatments <14% of the human co-players were fully cooperative during the last ten rounds of the game, as compared with >63% in the generosity treatments (see Supplementary Fig. 1). On the other extreme, a third of the human subjects refused to cooperate against an extortionate co-player during the last ten rounds of the game, whereas only 1 out of 30 subjects did so in the generosity treatments. Thus, although the different treatments provided similar monetary incentives for cooperation, subjects were more hesitant to cooperate against an extortionate co-player. Withholding cooperation against these ZD strategies can be considered as a form of costly punishment (Fig. 4 and Supplementary Fig. 2). For example, reducing one’s cooperation rate by 10% against strong extortioners decreased the opponent’s mean payoff per round by [euro] 0.029, but it also diminished the own payoff by [euro] 0.011. The resulting fine-to-cost ratio for punishment, 0.029/0.011≈2.6, is close to typical values used in experiments on costly punishment^32^. Being less cooperative thus led to a strong reduction in the co-player’s payoff, but it also turned out to be costly for the punishing individual itself.

## Discussion

Repeated games, and in particular the repeated prisoner’s dilemma, are model cases to explore the tension between cooperation and conflict in long-term social relations^33^. Although repetition was previously thought to promote cooperation, it has recently been suggested that iterated games may open the door for the systematic manipulation of opponents^19^. The newly discovered ZD strategies are surprisingly simple: they do not require to take the whole history of the game into account—it is sufficient to consider the last round only. Although previous literature on ZD strategies has focused on infinitely repeated games, social relationships in reality (and also our experiment) have a finite though fuzzy horizon. However, as we show in the Methods, this does not notably diminish the power of ZD strategies; if there is a sufficient number of rounds, ZD strategists have a similar amount of control as in the infinitely repeated game.

Two subclasses of ZD strategies have received particular attention: extortioners, as they are able to outcompete their direct opponents^19^, and generous ZD strategies, as they allow for stable mutual cooperation^25^,^26^. Herein, we have investigated the performance of these two strategy classes against human subjects. Our results confirm that extortioners dominated their direct opponents, but unexpectedly generosity turned out to be the more profitable strategy. In a way, extortion meant to ‘win each battle, but at the expense of losing the war’. These findings are superficially in line with previous evolutionary studies, which suggested that natural selection in well-mixed populations favours generous ZD strategies^26^,^27^. However, in these theoretical studies the success of generosity was based on a different argument; in an evolving population extortion does not prevail because mutual extortion is unstable, which leads extortioners to change their strategy^21^. In our experiment, the strategy of the extortioners was fixed, but extortioners were unable to motivate their co-players to cooperate fully, despite setting up appropriate incentives.

There are two possible explanations why humans were reluctant to cooperate against extortioners. On the one hand, subjects may have strived for high payoffs, but they did not have enough time to learn that they need to fully cooperate to reach this aim. This seems to be especially relevant as their opponents’ strategies were stochastic and thus not straightforward to predict. However, this argument does not explain why generous ZD players were more successful to catalyse cooperation than extortioners—after all, the implemented ZD strategies were equally complex and they provided comparable monetary incentives to promote cooperation. Instead, our results suggest that the subjects were not only driven by monetary considerations, but that they were willing to apply reciprocal strategies to oppose extortionate behaviours. In fact, several behavioural studies have reported that a large fraction of humans can be described as conditional cooperators^34^,^35^,^36^. In line with this hypothesis, we find that humans were almost four times more likely to cooperate in a given round if their co-player did so in the previous round (human cooperation rates were 81.1% if the co-player cooperated in the previous round, and 22.0% otherwise, see Supplementary Table 2). Reciprocal behaviours in turn can have various behavioural roots, such as conformism, or the wish to enforce fair outcomes^37^,^38^,^39^. In our generosity treatments the two possible objectives, payoff-maximization and fairness, were perfectly aligned; by maximizing their expected payoffs humans also ensured equal outcomes. In contrast, in the extortion treatments there was a trade-off; humans that aimed to maximize their payoffs had to accept the most unfair outcome. As more than half of the participants declared in the post-experiment questionnaire that equality motives affected their decisions, the wish to ensure fair outcomes may have been an important reason for the downfall of extortion.

However, unlike in other strategic situations as in the ultimatum game^40^, unfairness was not straightforward to detect in our behavioural experiment. It is not a single selfish decision that makes an opponent behaving extortionate. Rather, it is the systematic interplay of selfishness and cooperation, which only unfolds itself over the course of the game. At first sight, the extortionate strategies described by Press and Dyson^19^ look rather inconspicuous (which may be one of the reasons why these strategies were discovered only recently). Extortioners apply a simple, conditionally cooperative strategy—with a slight bias to their own advantage. Although this more implicit form of selfishness seems to be more difficult to detect, humans have evolved mechanisms such as conditional cooperation that prevent them from being exploited.

Although our experiment did not entail an explicit punishment option, we found that by withholding contributions, subjects applied an implicit form of costly punishment. Such an effect has not been reported previously. In fact, it seems difficult to show such an effect with a conventional experiment, in which two human subjects play against each other. One would have to demonstrate that withholding cooperation is indeed individually costly. However, this seems almost impossible, as long as the co-player’s strategy is unknown (for example, against an unconditional defector, withholding cooperation is the best response and hence no instance of costly punishment). Overall, our results thus suggest that sufficient monetary incentives alone are not enough to induce cooperation in long-term social relationships. Instead, humans take additional motives such as individual intentions and fairness considerations into account, and they are ready to fight back when they feel exploited.

## Methods

### Experimental design

Experiments were conducted in November and December 2013 at the universities of Kiel and Hamburg, Germany, with subjects recruited from a first-year course in biology. All participants gave their informed consent to participate. For each of ten experimental sessions, we invited six volunteers to participate in a game. To ensure the subjects’ anonymity, participants were separated by opaque partitions, they were playing under a neutral pseudonym and they were not allowed to talk to each other during or after the experiment. All experimental decisions were made on a computer screen using the experimental software Z-Tree^41^. As we were interested in the relative performance of extortionate and generous strategies, participants were not playing against each other, but against a randomly determined computer strategy (out of the four alternatives ES, EM, GM or GS, as outlined in Table 1). The subjects’ instructions were kept in a neutral way, that is, subjects were neither told that they would interact with a computer opponent nor that they would play against each other (see Supplementary Methods for a translation of the experiment’s instructions). The game consisted of 60 rounds of the prisoner’s dilemma (subjects were not informed about the exact duration of the game, but rather that they would play over many rounds). The experiment took ~1 h. Including the show-up fee of [euro] 10, individual earnings were on average between [euro] 17.65 (in the strong extortion treatment) and [euro] 26.78 (in the strong generosity treatment).

### Theoretical predictions

The extortionate and generous strategies used for the experiment are instances of a more general strategy class, the class of ZD strategies. In an infinitely repeated prisoner’s dilemma, a ZD strategist can unilaterally enforce a linear relation between his own payoff *π* and the co-player’s payoff 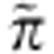. That is, payoffs obey a linear relation of the form^19^,^21^,^25^,^26^


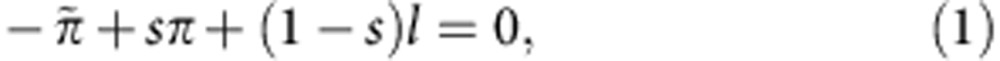


where *l* and *s* are characteristic properties of the applied ZD strategy^27^. The baseline payoff *l* can be interpreted as the payoff of a ZD strategy against itself (for the two extortionate strategies *l*=*P*, and for the two generous strategies *l*=*R*). The slope *s* determines how strongly the payoffs of the two players are correlated (for the two mild treatments, we have used *s*=2/3, corresponding to a rather high correlation; for the two strong treatments, we have used *s*=1/3). If the prisoner’s dilemma is only repeated for a finite number of rounds *M*, equation (1) does not need to be satisfied any longer. Nevertheless, one can derive the following estimate for the players’ expected payoffs (see Supplementary Methods),


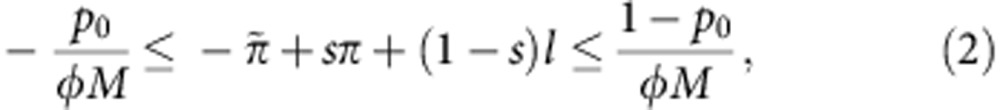


where *p*_0_ is the probability that the ZD-strategist cooperates in the first round and *φ* is a constant. In Fig. 2, the expected payoff range according to equation (2) is depicted as a thin black area. See Supplementary Methods for further details.

## Author contributions

C.H., T.R. and M.M. designed the research; C.H. and M.M. performed the experiment and wrote the paper.

## Additional information

**How to cite this article:** Hilbe, C. *et al.* Extortion subdues human players but is finally punished in the prisoner’s dilemma. *Nat. Commun.* 5:3976 doi: 10.1038/ncomms4976 (2014).

## Supplementary Material

Supplementary Information Supplementary Figures 1-2, Supplementary Tables 1-2, Supplementary Methods and Supplementary References

## Figures and Tables

**Table 1:** Overview of the experimental design.

**Treatment**	**Number of human co-players**	**Cooperation probabilities**					**Slope**
		***p***_**0**_	***p***_**R**_	***p***_**S**_	***p***_**T**_	***p***_**P**_	***s***
ES	16	0.000	0.692	0.000	0.538	0.000	1/3
EM	14	0.000	0.857	0.000	0.786	0.000	2/3
GM	14	1.000	1.000	0.077	1.000	0.154	2/3
GS	16	1.000	1.000	0.182	1.000	0.364	1/3

ES, strong extortion; EM, mild extortion; GM, mild generosity; GS, strong generosity; ZD, zero determinant.

In each of the four treatments, the computer played according to a different ZD strategy. ZD strategies are defined by five probabilities: *p*_0_ is the probability to cooperate in round *m*=1, and for *i*ε{*R*, *S*, *T*, *P*} the value of *p*_*i*_ is the probability to cooperate in round *m*>1 after receiving the payoff *i* in round *m*−1, see refs 6, 31. Extortionate strategies do not cooperate in the first round, and they never cooperate after mutual defection. Generous strategies, on the other hand, cooperate in the first round and they always cooperate after mutual cooperation. For a derivation of the implemented cooperation probabilities, we refer to the Supplementary Methods. The parameter *s* determines the slope of the predicted payoff relation: for example, a slope of *s*=2/3 implies that for each Cent that the ZD strategist earns additionally, the human co-player’s additional payoff is 2/3 Cents. In general, a smaller slope increases the payoff inequality between players: decreasing the value of *s* makes extortionate ZD strategies even more extortionate, whereas it makes generous ZD strategies even more generous. For this experiment, we followed the parameters of ref. 3, that is, the payoffs were set to *T*=[euro]0.50, *R*=[euro]0.30, *P*=[euro]0.10 and *S*=[euro]0.00.
