# Supplementary material for: Extortion subdues human players but is finally punished in the prisoner’s dilemma
Source: Nat Commun. 2014 May 29;5:3976. doi: 10.1038/ncomms4976 (PMC4050275; doi:10.1038/ncomms4976)
Supplement: Supplementary Information — Supplementary Figures 1-2, Supplementary Tables 1-2, Supplementary Methods and Supplementary References [file ncomms4976-s1.pdf]

Supplementary Figures

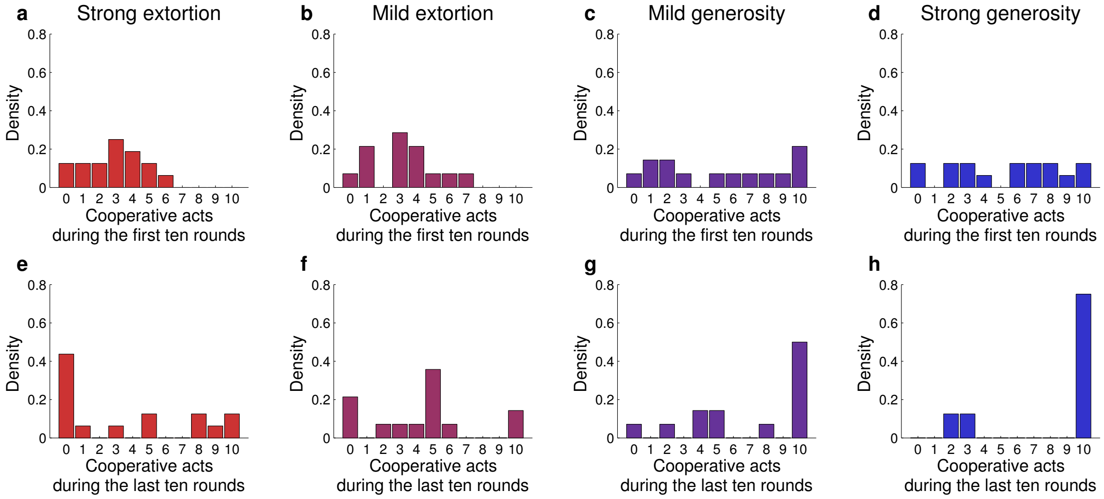

Supplementary Figure 1: Comparison between human cooperation rates in the beginning and in the end of the experiment. In each graph, the horizontal axis shows how often human subjects cooperated during the first ten rounds (**a - d**) or during the last ten rounds (**e - h**). In the beginning of the experiment, behaviors were rather evenly distributed. By the end of the experiment, however, most subjects either had a high cooperation rate (in the two generosity treatments) or they had a low or moderate cooperation rate (in the two extortion treatments). Nevertheless there were also a few subjects in the extortion treatments that were fully cooperative during the last ten rounds (2 out of 16 subjects in the strong extortion treatment, and 2 out of 14 subjects in the mild extortion treatment). In the post-experiment questionnaire, these cooperative subjects stated that they wanted to establish a regime of mutual cooperation, although most of them realized that their opponent was somewhat more selfish.

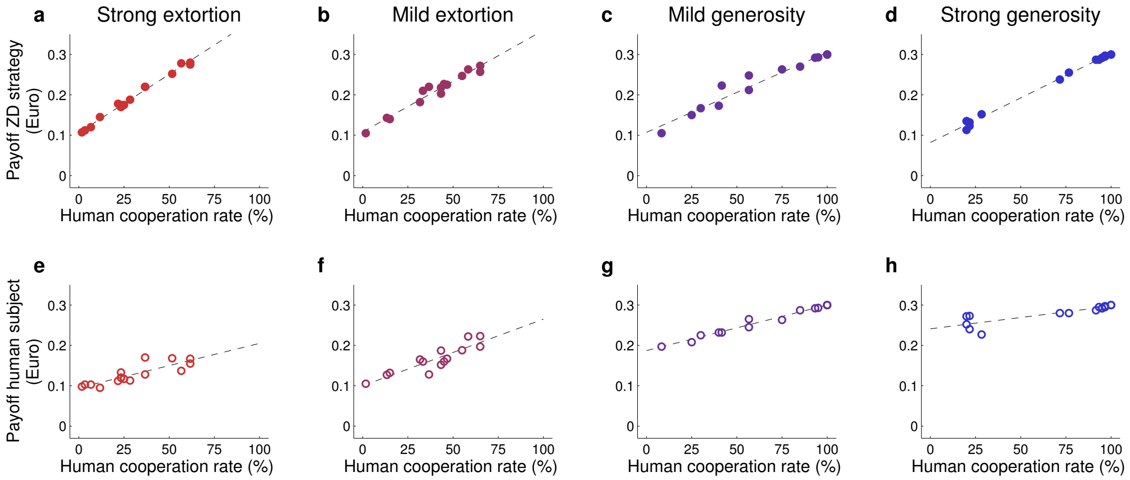

Supplementary Figure 2: Effects of human cooperation on the payoffs of ZD strategies (**a** - **d**) and on the human subjects' payoffs (**e** - **h**). In all graphs, the horizontal axis shows the fraction of rounds in which the human players cooperated. Colored dots represent the outcome of the experiment, whereas the dashed line depicts the linear regression curve based on a least squares analysis. (**a** - **d**) As expected, human cooperation had a positive impact on the payoffs of their ZD co-players: if humans increased their cooperation rate by 10 %, a linear regression analysis suggests that the payoffs per round of the ZD strategies increased by € 0.029 (ES), € 0.025 (EM), € 0.020 (GM), and € 0.022 (GS), respectively. (**e** - **h**) Also the human subjects themselves benefited from being more cooperative: an increase of their cooperation rates by 10 % resulted in an increase of their own payoffs by € 0.011 (ES), € 0.017 (EM), € 0.011 (GM), and € 0.006 (GS), respectively.

Supplementary Tables

Treatment		1	2	3	4	5	6	7	8	9	10	11	12	13	14	15	16	∅
Strong extortion	f	21	17	9	11	8	8	22	8	1	5	24	1	16	0	2	8	**10.1**
	$\tilde{f}$	31	34	14	22	14	15	37	17	2	13	37	7	22	1	4	14	**17.7**
	π	25.2	27.8	17.5	22.0	17.0	17.5	28.0	18.8	11.2	17.8	27.5	14.5	22.0	10.7	12.0	17.0	**19.2**
	$\tilde{\pi}$	16.8	13.7	13.3	12.8	12.0	11.7	15.5	11.3	10.3	11.2	16.7	9.5	17.0	9.8	10.3	12.0	**12.8**
Mild extortion	f	30	19	26	1	30	11	17	6	14	24	18	21	35	8	-	-	**18.6**
	$\tilde{f}$	39	27	33	1	35	22	19	8	20	26	26	28	39	9	-	-	**23.7**
	π	27.2	22.7	24.7	10.5	26.3	22.0	18.2	14.3	21.0	20.3	21.8	22.5	25.7	14.0	-	-	**20.8**
	$\tilde{\pi}$	19.7	16.0	18.8	10.5	22.2	12.8	16.5	12.7	16.0	18.7	15.2	16.7	22.3	13.2	-	-	**16.5**
Mild generosity	f	60	38	22	57	53	26	56	60	25	31	60	16	45	36	-	-	**41.8**
	$\tilde{f}$	60	34	15	57	51	25	56	60	18	24	60	5	45	34	-	-	**38.9**
	π	30.0	21.2	15.0	29.3	27.0	22.3	29.2	30.0	16.7	17.3	30.0	10.5	26.3	24.8	-	-	**23.5**
	$\tilde{\pi}$	30.0	24.5	20.8	29.3	28.7	23.2	29.2	30.0	22.5	23.2	30.0	19.7	26.3	26.5	-	-	**26.0**
Strong generosity	f	58	31	58	26	57	49	60	58	26	60	31	57	48	26	57	55	**47.3**
	$\tilde{f}$	58	13	58	13	57	46	60	58	17	60	12	56	43	12	57	55	**42.2**
	π	29.5	12.3	29.5	13.2	29.2	25.5	30.0	29.7	15.2	30.0	11.3	28.7	23.8	13.5	29.2	28.7	**23.7**
	$\tilde{\pi}$	29.5	27.3	29.5	24.0	29.2	28.0	30.0	29.7	22.7	30.0	27.2	29.5	28.0	25.2	29.2	28.7	**28.0**

Supplementary Table 1: Results of the experiment for each of the human-computer interactions of the experiment (16 interactions in each of the two strong treatments, and 14 interactions in each of the two mild treatments). The rows show the number of cooperative decisions for the computer (f) and for humans ($\tilde{f}$) over the sixty rounds of the experiments, as well as the resulting average payoffs per round in cents (π for the computer and $\tilde{\pi}$ for the human co-player, respectively).

Treatment	Decision of computer in previous period	Number of observations	Likelihood of human cooperation next round
Strong extortion	C	157	81.5%
	D	787	19.1%
Mild extortion	C	246	77.6%
	D	580	20.7%
Mild generosity	C	575	83.1%
	D	251	23.5%
Strong generosity	C	742	80.6%
	D	202	35.1%
Average over all treatments	C	1,720	81.1%
	D	1,820	22.0%

Supplementary Table 2: Evidence for conditional cooperation across treatments. Previous experiments on public good games suggest that humans are often conditionally cooperative, i.e. they wish to reciprocate the co-players' actions, see Refs. (1–3). Because generous ZD strategies are more cooperative than extortionate ZD strategies, this may explain why humans were significantly more cooperative in the generous treatments. In order to explore this explanation in more detail, we report how human subjects reacted to the outcome of the previous round. The third column shows the number of instances in which the computer cooperated during the first 59 rounds of the game (with C referring to cooperation and D referring to defection). The fourth row shows how often the human player cooperated in the following round. In all treatments, human cooperation rates were between 77.6% and 83.1% if the computer cooperated in the previous round. Otherwise, human cooperation rates were only between 19.1% and 35.1%.

Supplementary Methods

Theoretical Methods

Zero-determinant strategies in finitely repeated games

Previous studies on ZD strategies have considered infinitely repeated games (4–10), whereas the experiment had a finite number of rounds. We will demonstrate here that the definition of ZD strategies can be appropriately extended. Moreover, we will show that if the game is repeated sufficiently often, then the ZD strategies for the finitely repeated game allow a similar degree of control as in the original setup.

Let us consider a repeated prisoner's dilemma with possible payoffs R (if both players cooperate), S (if the focal player cooperates, and the co-player defects), T (if the focal player defects, and the co-player cooperates), and P (if both players defect). We assume that the typical relation $T > R > P > S$ holds. ZD strategies belong to the class of memory-one strategies: the decision whether to cooperate in a given round only depends on the outcome of the previous round. Such strategies can be written as a 5-tuple $(p_0, p_R, p_S, p_T, p_P)$. In this representation, p_0 is the player's probability to cooperate in round $m = 1$, and p_i for $i \in \{R, S, T, P\}$ is the probability to cooperate in round $m \geq 2$ after obtaining the payoff i in round $m - 1$. ZD strategies are defined as those memory-one strategies for which there are constants l, s, and $\phi > 0$ such that

$$\begin{aligned}
p_R &= 1 - \phi(1-s)(R-l) \\
p_S &= 1 - \phi\big[(1-s)(S-l) + T - S\big] \\
p_T &= \phi\big[(1-s)(l-T) + T - S\big] \\
p_P &= \phi(1-s)(l-P)
\end{aligned} \tag{1}$$

(this definition follows from the definition given by Press and Dyson, Ref. (4), by using the transformation $\alpha = \phi s$, $\beta = -\phi$, and $\gamma = \phi(1-s)l$). The following property of ZD strategies is central to our experiment:

Proposition 1 (An estimate for ZD strategies)
Consider a finitely repeated prisoner's dilemma with M rounds. Suppose one player applies a ZD strategy $(p_0, p_R, p_S, p_T, p_P)$ with parameters l, s, and $\phi > 0$. Let π denote the resulting average payoff per round for the ZD strategist, and let $\tilde{\pi}$ denote the respective payoff of the

co-player. Then independent of the co-player's strategy, payoffs satisfy the relation

$$-\frac{p_0}{\phi M} \;\leq\; (1-s)l + s\pi - \tilde{\pi} \;\leq\; \frac{1-p_0}{\phi M}. \tag{2}$$

In particular, it follows that

$$\big|(1-s)l + s\pi - \tilde{\pi}\big| \leq \frac{1}{\phi M}. \tag{3}$$

Proof. The proof is merely a slight variation of the proofs presented in Ref. (5) and Ref. (10). For $i \in \{R,S,T,P\}$ let $v_i(m)$ denote the probability that the payoff of the ZD strategist in round m is i. Let us introduce the following vector notation:

$$\begin{aligned}
\mathbf{v}(m) &= \big(v_R(m), v_S(m), v_T(m), v_P(m)\big)^{\mathsf{T}}\\
\mathbf{p} &= \big(p_R, p_S, p_T, p_P\big)\\
\mathbf{g} &= \big(R, S, T, P\big)\\
\tilde{\mathbf{g}} &= \big(R, T, S, P\big)\\
\mathbf{1} &= \big(1,1,1,1\big)\\
\mathbf{e} &= \big(1,1,0,0\big)
\end{aligned}$$

Using this notation, we can write the ZD strategist's expected payoff in round m as $\pi(m) = \mathbf{g}\cdot\mathbf{v}(m)$, and the co-player's expected payoff in that round as $\tilde{\pi}(m) = \tilde{\mathbf{g}}\cdot\mathbf{v}(m)$. Additionally, the definition of ZD strategies, Eq. (1), implies the identity

$$\mathbf{p} = \mathbf{e} + \phi\big[(1-s)(l\mathbf{1} - \mathbf{g}) + \mathbf{g} - \tilde{\mathbf{g}}\big]. \tag{4}$$

Let $q(m)$ denote the ZD strategist's probability to cooperate in round m. Using the previous notation, we can write $q(m) = \mathbf{e}\cdot\mathbf{v}(m)$ and $q(m+1) = \mathbf{p}\cdot\mathbf{v}(m)$. Thus, the quantity $w(m) := q(m+1) - q(m)$ satisfies the relation

$$w(m) = (\mathbf{p}-\mathbf{e})\cdot\mathbf{v}(m) = \phi\big[(1-s)(l\mathbf{1}-\mathbf{g}) + \mathbf{g} - \tilde{\mathbf{g}}\big]\cdot\mathbf{v}(m) = \phi\big[(1-s)l + s\pi(m) - \tilde{\pi}(m)\big] \tag{5}$$

Taking the definition of $w(m)$, it follows that

$$\frac{1}{M}\sum_{m=1}^{M} w(m) = \frac{q(M+1)-q(1)}{M} = \frac{q(M+1)-p_0}{M} \tag{6}$$

On the other hand, by Eq. (5) we have

$$\frac{1}{M}\sum_{m=1}^{M} w(m) = \frac{\phi}{M}\sum_{m=1}^{M}\big[(1-s)l + s\pi(m) - \tilde{\pi}(m)\big] = \phi\big[(1-s)l + s\pi - \tilde{\pi}\big]. \qquad (7)$$

As the two expressions (6) and (7) need to coincide, and as the definition of $q(m)$ requires $0 \le q(m) \le 1$, it follows that

$$-\frac{p_0}{\phi M} \;\le\; (1-s)l + s\pi - \tilde{\pi} \;\le\; \frac{1-p_0}{\phi M}. \qquad \square$$

We note that in the limit of infinitely repeated games, $M \to \infty$, we recover the result that ZD strategies enforce a linear relationship between payoffs,

$$\tilde{\pi} = s\pi + (1-s)l. \qquad (8)$$

By choosing appropriate parameters l, s, and ϕ, the ZD strategist has a direct influence on this functional relationship.

Derivation of the strategies used for the experiment

Our experiment considers the performance of four different ZD strategies. To choose the corresponding parameters p_0, l, s and ϕ of these ZD strategies, we applied the following considerations:

- Parameter p_0: As extortioners are defined as strategies that cannot be outperformed by any opponent, they need to set $p_0 = 0$ (otherwise they would be outperformed by unconditional defectors). Analogously, a generous strategy needs to set $p_0 = 1$.

- Parameter l: Due to a similar reasoning, extortionate strategies require $l = P$, see Refs. (4,9), whereas generous ZD strategies require $l = R$, see Refs. (7,9).

- Parameter s: Since both strategy classes, extortioners and generous ZD strategies, require s to be in the unit interval $0 \le s \le 1$, Refs. (4,11), we have chosen to use $s = 1/3$ (for the two "strong" treatments) and $s = 2/3$ (for the two "mild" treatments).

- Parameter ϕ: According to inequality (3), higher values of ϕ allow a ZD strategist to have a better control over the resulting payoff relations. Thus, in order to reduce the variance of our results, we have used the maximum ϕ-value (subject to the constraint that the resulting probabilities in Eq. (1) need to satisfy $0 \le p_i \le 1$, for all i).

Using the payoff values of the experiment $T = 0.5$, $R = 0.3$, $P = 0.1$ and $S = 0.0$, and Eq. (1), these parameter choices imply the following ZD strategies for the experiment (see also Table 1 in the main text):

Strong extortion ($l = P$, $s = 1/3$, $\phi = 30/13$)

$$p_0 = 0.000, \quad p_R = 0.692, \quad p_S = 0.000, \quad p_T = 0.538, \quad p_P = 0.000.$$

Mild extortion ($l = P$, $s = 2/3$, $\phi = 15/7$)

$$p_0 = 0.000, \quad p_R = 0.857, \quad p_S = 0.000, \quad p_T = 0.786, \quad p_P = 0.000.$$

Mild generosity ($l = R$, $s = 2/3$, $\phi = 30/13$)

$$p_0 = 1.000, \quad p_R = 1.000, \quad p_S = 0.077, \quad p_T = 1.000, \quad p_P = 0.154.$$

Strong generosity ($l = R$, $s = 1/3$, $\phi = 30/11$)

$$p_0 = 1.000, \quad p_R = 1.000, \quad p_S = 0.182, \quad p_T = 1.000, \quad p_P = 0.364.$$

Predictions for the experiment

In the experiment, the prisoner's dilemma was played for $M = 60$ rounds. Therefore, the inequalities in (2) predict the following relation between the expected payoff π of the ZD strategist and the expected payoff $\tilde{\pi}$ of the human co-player:

Strong extortion ($p_0 = 0$, $l = P = 0.1$, $s = 1/3$, $\phi = 30/13$)

$$\frac{1}{3} \cdot \pi + \frac{2}{3} \cdot 0.1 - \frac{13}{1800} \quad \leq \quad \tilde{\pi} \quad \leq \quad \frac{1}{3} \cdot \pi + \frac{2}{3} \cdot 0.1 \tag{9}$$

Mild extortion ($p_0 = 0$, $l = P = 0.1$, $s = 2/3$, $\phi = 15/7$)

$$\frac{2}{3} \cdot \pi + \frac{1}{3} \cdot 0.1 - \frac{7}{900} \quad \leq \quad \tilde{\pi} \quad \leq \quad \frac{2}{3} \cdot \pi + \frac{1}{3} \cdot 0.1 \tag{10}$$

Mild generosity ($p_0 = 1$, $l = R = 0.3$, $s = 2/3$, $\phi = 30/13$)

$$\frac{2}{3} \cdot \pi + \frac{1}{3} \cdot 0.3 \quad \leq \quad \tilde{\pi} \quad \leq \quad \frac{2}{3} \cdot \pi + \frac{1}{3} \cdot 0.3 + \frac{13}{1800} \tag{11}$$

Strong generosity ($p_0 = 1$, $l = R = 0.3$, $s = 1/3$, $\phi = 30/11$)

$$\frac{1}{3} \cdot \pi + \frac{2}{3} \cdot 0.3 \quad \leq \quad \tilde{\pi} \quad \leq \quad \frac{1}{3} \cdot \pi + \frac{2}{3} \cdot 0.3 + \frac{11}{1800} \tag{12}$$

In Fig. 2 of the main text, we have illustrated these inequalities: the pairs $(\pi,\tilde{\pi})$ that satisfy the above constraints are shown as black lines (note that the estimates (9) – (12) are statements about expected payoffs. As ZD strategies are stochastic, and as the game is only repeated for a finite number of rounds, realized payoffs do not need to satisfy the above inequalities, as can be seen in Fig. 2.) For the two extortionate treatments, these black lines are on or below the diagonal, implying that humans never yield a higher expected payoff than their extortionate co-players. Analogously, in the two generous treatments, the black lines are on or above the diagonal, implying that humans are never worse off than their generous co-players.

If human subjects aim to maximize their payoffs, their best response in all four treatments is to cooperate in all rounds. In fact, as the ZD strategist enforces a positive relation between payoffs ($s > 0$), subjects maximize their own payoff $\tilde{\pi}$ by maximizing their co-player's payoff π. When humans play their best response, expected payoffs can be calculated as

	Payoff π of ZD strategist	Payoff $\tilde{\pi}$ of human co-player
Strong extortion (ES)	0.37	0.19
Mild extortion (EM)	0.33	0.25
Mild generosity (GM)	0.30	0.30
Strong generosity (GS)	0.30	0.30

(13)

Thus, the payoffs of the ZD strategies satisfy $\pi_{ES} > \pi_{EM} > \pi_{GM} = \pi_{GS}$ – if human subjects move towards full cooperation in all four treatments, then extortionate ZD strategies should receive higher payoffs than their generous counterparts. On the other hand, for the human co-players we obtain the relation $\tilde{\pi}_{GS} = \tilde{\pi}_{GM} > \tilde{\pi}_{EM} > \tilde{\pi}_{ES}$ – not very surprisingly, humans prefer their opponents to be generous, rather than extortionate.
In summary, we have the following predictions:

1. Independent of how human subjects play, extortionate ZD strategies get at least the payoff of their co-players in each game. Generous ZD strategies obtain at most the payoff of their opponent.

2. Assuming that human subjects aim to maximize their payoffs, we would expect that their cooperation rates increase over the course of the game.

3. If there is a comparable trend towards cooperation across treatments, extortionate ZD strategies should earn higher payoffs than generous ZD strategies.

In response to our theoretical predictions, the main text reports the following findings:

1. In line with the theoretical prediction, the two extortionate strategies gained higher payoffs than their human co-players, whereas the two generous strategies obtained lower payoffs than their co-players.
2. Only in the generous treatments humans became significantly more cooperative over the course of the game (cooperation rates increased from 53 % during the first ten rounds to 76 % during the last ten rounds of the experiment, Wilcoxon matched-pairs signed-rank test, $n_G = 30$, $Z = 3.161$, $p = 0.002$). In the extortionate treatments there was only a slight trend towards more cooperation, and this trend failed to be significant (cooperation rates increased from 30.3 % to 39.7 %, Wilcoxon matched-pairs signed-rank test, $n_E = 30$, $Z = 1.131$, $p = 0.258$).
3. In contrast to our prediction, generous strategies obtained higher payoffs than extortioners ($\pi_G =$ € 0.236 vs. $\pi_E =$ € 0.199, $n_E = n_G = 30$, $Z = -2.544$, $p = 0.011$), because human subjects were more cooperative in the generous treatments (human cooperation rates were 67.7 % against generous strategies and only 34.2 % against extortioners, Mann-Whitney U-test, $n_E = n_G = 30$, $Z = -3.625$, $p < 0.001$).

Experimental Methods

The experiment was conducted in November and December 2013, at the University of Kiel and at the University of Hamburg, Germany. As participants, we have recruited 60 volunteers from first-year courses in Biology. These volunteers participated in ten groups of six subjects each in a computerized experiment. Before each experimental session, subjects were orally informed by one of the experimenters (M. M.) about how to operate the computers, and about the measures that were taken to ensure the subjects' anonymity. These measures included that subjects made their decisions under a neutral pseudonym, and that subjects were not allowed to talk to each other during or after the experiment. Moreover, they were informed that they would receive all their earnings in cash after the experiment. The payment procedure was organized in a way such that the anonymity of the participants was fully maintained.

After these oral instructions, participants were randomly assigned to a seat. The seats were separated by opaque partitions. Each seat came with a laptop computer, which informed participants about the rules of the game, and with which participants could communicate their decisions. The game instructions did not reveal the nature of the subjects' opponents: subjects were not told that they would play against each other, but they were also not told that they would play against a computer program (for a translation of the instructions, see Section 3). Participants were unaware of the exact length of the game; they were only informed that they would interact with their respective co-player over many rounds and that no time limit

existed for their decisions to be done.

The computer opponents were randomly assigned to the six different seats. In order to avoid sequence effects, we ensured that in all experimental sessions there was at least one instance of each of the four computer strategies, ES, EM, GM, and GS. As human subjects played independently of each other, we considered each subject-computer interaction as the statistical unit of the experiment. For our analysis, we have used two-tailed tests throughout.

Instructions of the experiment

In the following, we provide the information displayed on the subjects' laptops throughout the experiment, translated from German. The instructions were the same for all four treatments.

Instructions in the beginning of the experiment

Page 1. Welcome to this experiment, in which you can earn money. At the beginning of this experiment, you will receive 10 Euros credited to your account. During the experiment you can earn more money. This may depend on your own decision and on the decision of others. Your decisions are anonymous. To ensure this, the computer assigns you a pseudonym that can be seen at the bottom left of your screen. The pseudo names are names of moons in our solar system (Ananke, Telesto, Despina, Japetus, Metis, and Kallisto). At the end of the game you will receive in cash the money in your account anonymously under your pseudo name. To render this experiment successful, it is strictly forbidden for participants to talk to each other or to communicate in any other way. After having read this text completely, please confirm by the pressing the OK-button.

Page 2. In the beginning of this experiment the computer will assign you a random co-player. Your co-player will remain the same for the whole experiment. The experiment consists of several rounds. In each round, you and your co-player face the same decision situation. An explanation of this decision situation follows on the next page. After having read this text completely, please confirm by pressing the OK-button.

Page 3. In each round, both players need to simultaneously choose a letter (either **C** or **D**). Each player needs to decide without knowing the choice of the co-player. Your payoff depends on your decision and on the decision of your co-player. The following table shows all possible payoffs. The first amount in each cell (with a blue font color) is your own payoff, and the second amount (with a green font color) is the payoff of your

co-player:

		Decision of your co-player C	D
Your	**C**	€ 0.30, € 0.30	€ 0.00, € 0.50
decision	**D**	€ 0.50, € 0.00	€ 0.10, € 0.10

So there are four possible outcomes:

You: **C**	Your co-player: **C**	You get € 0.30	Your co-player gets € 0.30
You: **C**	Your co-player: **D**	You get € 0.00	Your co-player gets € 0.50
You: **D**	Your co-player: **C**	You get € 0.50	Your co-player gets € 0.00
You: **D**	Your co-player: **D**	You get € 0.10	Your co-player gets € 0.10

After having read this text completely, please confirm by pressing the OK-button.

Page 4. In each round you will have to answer the same question: "Which letter do you choose (either **C** or **D**)?" You decide by clicking on the corresponding button. Then you need to confirm your decision by clicking on the OK-button. You can only see the outcome of this round if all players have clicked on the OK-button. After having read this text completely, please confirm by pressing the OK-button.

Page 5. Examples: In each round you and your co-player are asked "Which letter do you choose (either **C** or **D**)?". After you have independently chosen a letter, the outcome of this round is displayed.

Example 1:

You: **C** Your co-player: **C** You get € 0.30 Your co-player gets € 0.30

Example 2:

You: **D** Your co-player: **C** You get € 0.50 Your co-player gets € 0.00

Example 3:

You: **D** Your co-player: **D** You get € 0.10 Your co-player gets € 0.10

Players need to confirm that they have read this page with the results by clicking on the OK-button. Then the round is over, and both players receive their payoffs credited to their account. The experiment consists of many rounds. In each round you face the same decision situation (but you can decide differently in each round). After having read this text completely, please confirm by pressing the OK-button.

Page 6. The experiment starts now! You have a credit of 10 Euros on your account. After having read this text completely, please confirm by pressing the green OK-button.

Instructions during the experiment

Page 1. Payoff table:

		Decision of your co-player	
		**C**	**D**
Your	**C**	€ 0.30, € 0.30	€ 0.00, € 0.50
decision	**D**	€ 0.50, € 0.00	€ 0.10, € 0.10

Which letter do you choose (either **C** or **D**)?

After having made your decision, please confirm by pressing the OK-button.

Page 2. Outcome of this round:

You: **X** Your co-player: **Y** You get € x Your co-player gets € y

After having read this text completely, please confirm by pressing the green OK-button.

Supplementary References

1. Claudia Keser and Frans van Winden. Conditional cooperation and voluntary contributions to public goods. *Scandinavian Journal of Economics*, 102:23–39, 2000.

2. Urs Fischbacher, Simon Gächter, and Ernst Fehr. Are people conditionally cooperative? evidence from a public goods experiment. *Economic Letters*, 71:397–404, Apr 2001.

3. Urs Fischbacher and Simon Gächter. Social preferences, beliefs, and the dynamics of free riding in public goods experiments. *American Economic Review*, 100(1):541–556, 2010.

4. W. H. Press and F. D. Dyson. Iterated prisoner's dilemma contains strategies that dominate any evolutionary opponent. *Proceedings of the National Academy of Sciences USA*, 109:10409–10413, 2012.

5. C. Hilbe, M. A. Nowak, and K. Sigmund. The evolution of extortion in iterated prisoner's dilemma games. *Proceedings of the National Academy of Sciences USA*, 110:6913–6918, 2013.

6. Ethan Akin. Stable cooperative solutions for the iterated prisoner's dilemma. *arXiv*, page 1211.0969v2, 2013.

7. A. J. Stewart and J. B. Plotkin. From extortion to generosity, evolution in the iterated prisoner's dilemma. *Proceedings of the National Academy of Sciences USA*, 110:15348–15353, 2013.

8. C. Adami and A. Hintze. Evolutionary instability of zero-determinant strategies demonstrates that winning is not everything. *Nature Communications*, 4:2193, 2013.

9. C. Hilbe, M. A. Nowak, and A. Traulsen. Adaptive dynamics of exortion and compliance. *PLoS One*, 8:e77886, 2013.

10. C. Hilbe, A. Traulsen, and K. Sigmund. Partners or rivals? Strategies for the iterated prisoner's dilemma. *Working Paper*, 2013.

11. A. J. Stewart and J. B. Plotkin. Extortion and cooperation in the prisoner's dilemma. *Proceedings of the National Academy of Sciences USA*, 109:10134–10135, 2012.
